# Development of Light-Scribing Process Using L-Ascorbic Acid for Graphene Micro-Supercapacitor

**DOI:** 10.3390/mi15070858

**Published:** 2024-06-30

**Authors:** Seorin Park, Da Young Lee, Sunghun Cho

**Affiliations:** School of Chemical Engineering, Yeungnam University, Gyeongsan 38541, Republic of Korea

**Keywords:** light-scribing, graphene, micro-supercapacitor, micro-patterning, l-ascorbic acid

## Abstract

The rapid development of smart technologies is accelerating the growing demand for microscale energy storage devices. This work reports a facile and practical approach to fabricating interdigitated graphene micro-patterns through the LSC process accompanied by the l-ascorbic acid (L-AA) and preheating treatment. Our work offered a higher degree of GO reduction than the conventional microfabrication. It significantly shortened the overall processing time to obtain the micro-patterns with improved electrical and electrochemical performances. The interdigitated MSC composed of 16 electrodes exhibited a high capacitance of 14.1 F/cm^3^, energy density of 1.78 mWh/cm^3^, and power density of 69.9 mW/cm^3^. Furthermore, the fabricated MSC device demonstrated excellent cycling stability of 88.2% after 10,000 GCD cycles and a high rate capability of 81.1% at a current density of 1.00 A/cm^3^. The fabrication process provides an effective means for producing high-performance MSCs for miniaturized electronic devices.

## 1. Introduction

Recently, there has been a rapid increase in interest in two-dimensional (2D) materials such as graphene, transition dichalcogenides, boron nitride (BN), Mxenes, and van der Waals heterostructures. These materials demonstrate unique electrical and optical properties, and their technological applications are expanding [1,2,3,4,5,6,7]. Among these 2D materials, graphene, a *sp*^2^-hybridized carbon nanomaterial with a one-atom-thick planar structure and a large specific surface area of 2640 m^2^/g, provides outstanding electron mobility, flexibility, and thermal and mechanical properties [6,7,8,9,10,11,12]. Since graphene enables energy storage through the EDLC mechanism, it has been widely used as a promising material for supercapacitors. In particular, the light-scribing (LSC) process is the most practical and innovative means for processing graphene into a micro-supercapacitor (MSC) of desirable size and shape [11,12,13,14,15]. This is because electrically conductive graphene micro-patterns can be mass-produced easily within a short time simply by attaching a flexible substrate coated with graphene oxide (GO) on a DVD and then inserting it into a DVD writer. In particular, the LSC process is differentiated from previous graphene manufacturing processes in that it enables site-specific reduction of GO [11,12,13,14,15]. Kaner et al. reported the LSC process to produce interdigitated micro-patterns with different sizes and shapes through the site-specific reduction of GO for the first time [11]. Previous studies on the LSC process of metal/reduced graphene oxide (RGO) composites for enhancing graphene micro-patterns’ electrical and electrochemical properties have been developed [13,14,15].

Despite the advantages of the LSC process mentioned above, the LSC method has several disadvantages. First of all, since the intensity of the 788 nm laser, which is the light source of the DVD writer, is not strong, it is difficult to achieve the desired degree of GO reduction with only one LSC process [10,11,12,13,14,15]. For this reason, repeating the LSC process several times or more is often necessary to realize the desired electrical and electrochemical properties. In addition, even if the LSC process is performed using the same DVD writer, deviations in the surface resistance and electrochemical activity of the micro-patterns inevitably occur depending on the deviations in the physical properties of the GO used. Recently, the eco-friendly reduction process of GO using green reducing agents, including l-ascorbic acid, metallic zinc, and sugar, has attracted great interest [16,17]. The reason is that these reducing agents are less harmful to the human body and are effective in converting GO into conductive RGO. In particular, L-AA has a photosensitive property that is oxidized when stimulated by UV–visible light, and the electrons released when L-AA is oxidized effectively reduce GO’s oxygenated functionalities [16]. Since this photosensitive oxidation reaction of L-AA works well even in a 788 nm light source generated from a DVD burner, the overall reduction efficiency of graphene through the LSC process is improved. In addition, the presence of L-AA is considered to shorten significantly the time required for the pre-reduction process of GO. Although several studies on the green reduction of GO using L-AA have been reported, studies applying the advantages of L-AA in the LSC process have not been reported. Therefore, developing the LSC process in the presence of L-AA is worthwhile for optimizing the conditions to produce high-quality graphene micro-patterns and MSCs within a shorter time.

Considering the problems described above, we report the facile fabrication of interdigitated graphene MSCs based on a modified LSC process in the presence of L-AA. The novelty of this study is that the reduction effect by heat treatment and the reduction effect by L-AA are incorporated into the LSC process for the first time. Graphene micro-patterns with an improved degree of reduction were obtained through these synergistic effects. When preheating GO for 15 min in the presence of 0.6 M L-AA, the degree of reduction of micro-patterns, electrical properties, and electrochemical properties was maximized after the LSC process. Owing to the synergistic effects of the L-AA and the LSC process, the MSC device obtained from our method demonstrated a high specific capacitance of 14.1 F/cm^3^, energy density of 1.78 mWh/cm^3^, power density of 69.9 mW/cm^3^, and retention rate of 88.2% after 10,000 GCD cycles.

## 2. Materials and Methods

### 2.1. Materials

Graphite flake, sodium nitrate (NaNO_3_, 99%), and polyvinyl alcohol (PVA, 99%, Mw: 89,000–124,000) were purchased from Sigma-Aldrich (St. Louis, MO, USA). Potassium permanganate (KMnO_4_, 99.3%) was purchased from Junsei Chemical Co. (Tokyo, Japan). Phosphorus pentoxide (P_2_O_5_, extra pure) and potassium persulfate (K_2_S_2_O_8_, 99%) were purchased from Kanto Chemical Co. (Tokyo, Japan). Phosphoric acid (H_3_PO_4_, 85%), sulfuric acid (H_2_SO_4_, 95%), hydrogen peroxide (H_2_O_2_, 30~35.5%), hydrochloric acid (HCl, 35~37%), and *N*-methylpyrrolidone (NMP, 99.7%) were purchased from Daejung Chemical & Metals Co., Ltd. (Siheung, Republic of Korea). Silver paste was purchased from CANS (Elcoat P-100, Tokyo, Japan).

### 2.2. Preparation of GO

The GO was created using a modified Hummer’s method [10,11,18]. To pre-oxidize the graphite flakes, 5 g of graphite powder, 2.5 g of P_2_O_5_, and 2.5 g of K_2_S_2_O_8_ were mixed with 30 mL H_2_SO_4_ and heated at 80 °C for 6 h. The resulting solution was filtered through a cellulose acetate filter (Whatman Inc., Clifton, NJ, USA) and washed with excess distilled water. The pre-oxidized graphite powders were then combined with 2.5 g of NaNO_3_ in 120 mL of H_2_SO_4_ and stirred vigorously for 30 min in an ice bath. A total of 15.0 g of KMnO_4_ was slowly added to the mixture over 45 min at a temperature of less than 20 °C, and the resulting mixture was heated at 35 °C for 4 h to obtain a gray–brown paste. After adding 700 mL of distilled water and 25 mL of H_2_O_2_ solution, the paste changed to a bright yellow color. The bright yellow paste was washed several times with 5 wt% HCl and distilled water to adjust the solution’s pH to 7. The solution was then sonicated to exfoliate the graphite oxide into graphene oxide (GO) and centrifuged at 4000 rpm to remove any residues. The as-prepared GO was dispersed in distilled water to create aqueous GO dispersions with a concentration of 2.0 mg/mL.

### 2.3. Preparation of GO Paste in the Presence of L-AA

The as-prepared aqueous dispersions of GO (2.0 mg/mL) were re-dispersed into a mixture of water and NMP (1:1, *v*/*v*). Then, 5 mL of 0.1, 0.2, 0.4, or 0.6 M L-ascorbic acid (L-AA) was added to the GO solution. Each sample containing different amounts of L-AA was sonicated for 30 min and then subjected to heat treatment at 90 °C for 15 min.

### 2.4. Preparation of GO Paste Adjusting the Heating Time

As-prepared aqueous dispersions of GO (2.0 mg/mL) were re-dispersed into a mixture of water/NMP (1:1, *v*/*v*), and each sample was sonicated for 30 min, followed by heat treatment at 90 °C for 1, 2, 4, 8, and 16 h, respectively.

### 2.5. Design of Interdigitated Micro-Pattern

**Figure 1:**
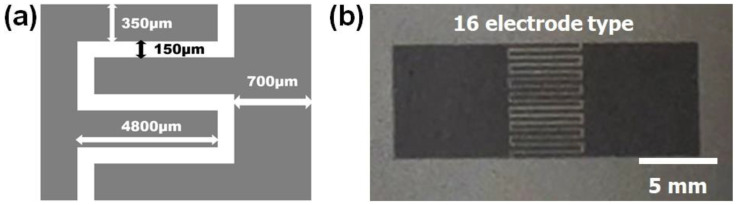
(**a**) Dimensions of the interdigitated micro-pattern obtained through LSC and (**b**) an actual digital image of interdigitated micro-pattern with 16 electrodes obtained through LSC.

This work proposed an interdigitated electrode consisting of 16 electrodes, with 8 positive and 8 negative electrodes. Each electrode was designed with a width of 350 μm and an interval between electrodes of 150 μm (Figure 1a,b). The total area and volume of the graphene micro-patterns were 37.87 mm^2^ and 0.227 mm^3^, respectively.

### 2.6. Fabrication of LSC Graphene Micro-Patterns

A PET sheet was cut to the size of a DVD and then glued onto a DVD media disc. Next, 5 g of GO paste was coated on the PET substrate and left to dry overnight at room temperature. The resulting GO film had an average thickness of about 6 μm. The GO-coated DVD was inserted into the DVD burner (Super-Multi GH-22LS30, LG Electronics Inc., Seoul, Republic of Korea) for the LSC process, which took approximately 20 min to create graphene micro-patterns. This LSC process was repeated 5 times, resulting in the creation of multiple micro-patterns with 16 interdigitated microelectrodes. In our experimental conditions, each DVD disc produced a total of 18 micro-electrodes. These micro-patterns were then used as MSC devices after an electrolyte layer was deposited onto them.

### 2.7. Preparation of PVA/H_2_SO_4_ Gel Electrolyte

A total of 1 g of PVA powder was added to 10 mL of distilled water, and the mixture was vigorously stirred at 90 °C until the PVA was dissolved entirely [13]. The PVA solution was cooled to 25 °C, followed by the addition of 1 g of H_2_SO_4_. The mixture solution was stirred vigorously until the homogeneous PVA/H_2_SO_4_ gel was obtained.

### 2.8. Fabrication of LSC-MSCs

A PVA/H_2_SO_4_ gel electrolyte was deposited on the active area of the interdigitated micro-pattern, and the excess solvent was evaporated by drying overnight at room temperature. The silver paste was coated to the edges of the microelectrode to ensure good electrical contact between the MSC devices and the electrochemical workstation.

### 2.9. Characterization of Graphene Micro-Patterns and LSC-MSCs

We used an FE-SEM (S-4800, HITACHI, LTD., Hitachi, Ibaraki, Japan) to study the morphology of graphene micro-patterns after the LSC process. The chemical bonds of the micro-patterns were investigated using a Fourier-transform infrared (FT-IR) spectrometer (Frontier, PerkinElmer Inc., Waltham, MA, USA). Ultraviolet–visible (UV-visible) spectra of the micro-patterns were measured using a UV-visible spectrometer (Mega-U600, Scinco Inc., Seoul, Korea). Raman spectra of the micro-patterns were recorded on a Micro-Raman spectrophotometer (XploRA, Horiba Scientific Inc., Kyoto, Japan). Surface resistivities of the micro-patterns were measured using a 4-point probe conductivity meter (Mode Systems Co., Hanam, Republic of Korea) equipped with a current source meter (Keithley 2400, Keithley Co., Cleveland, OH, USA). The electrochemical characteristics of the assembled MSC devices were investigated using an electrochemical workstation (ZIVE SP2, Wonatech, Seoul, Republic of Korea). Cyclic voltammetry (CV) curves of the MSCs were recorded in the voltage range 0–1.0 V at scan rates from 10 to 100 mV/s. GCD experiments were conducted by applying the 0 to 1.0 V voltages at current densities from 0.10 to 1.00 mA/cm^3^. Since the PVA/H_2_SO_4_ gel contains aqueous H_2_SO_4_, the stable voltage window for a single device should be within ~1.23 V. Therefore, the voltage window for a single MSC device was set to 1.0 V. However, when multiple devices are connected, the voltage window can be expanded up to ~4 V [10,11,12]. In the following data, the symbols *I*, Δ*t*, Δ*V*, and *v* denote the current, discharging time, potential window, and electrode volume, respectively. The volumetric capacitance *C_v_*, with units F/cm^3^, was calculated as *C_v_* = *I*Δ*t*/*v*Δ*V* [10,11,12,13,14,15]. The volumetric energy density, *E*, with units mWh/cm^3^, was calculated as *E* = *C_v_*Δ*V*^2^/2. The volumetric power density, *P*, with units mW/cm^3^, was calculated as *P* = *E*/*t*. The electrochemical impedance spectroscopy (EIS) characteristics of the MSCs were recorded in the frequency range from 1 MHz to 10 mHz.

## 3. Results

**Figure 2:**
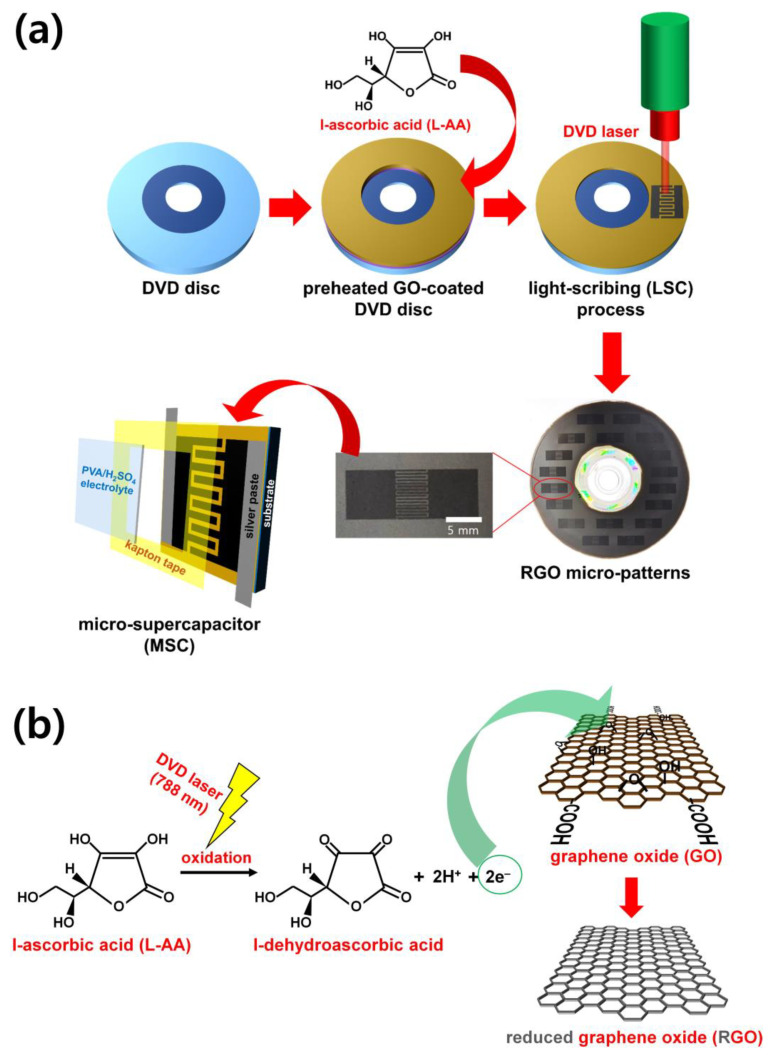
(**a**) A schematic diagram of the reduction process of graphene oxide using L-AA in LSC. (**b**) A schematic diagram of the reduction process of graphene oxide using L-AA in LSC.

Figure 2 shows the LSC processes and principles optimized in this study. In the first step, GO containing L-AA was heat treated for 15 min and then coated on a flexible substrate to prepare a GO film (Figure 2a). Then, through a heat-treatment process for about 15 min, a GO film was obtained, in which reduction and deoxygenation of GO were limitedly activated. The thickness of the GO film obtained here was about 6 μm. In the next step, LSC was performed by attaching the flexible substrate on which the GO film was deposited on a DVD and inserting it into a DVD burner. The critical point of the improved LSC process proposed in this study is the introduction of L-AA, which is environmentally friendly and photosensitive, into the LSC process. When a 788 nm laser light source stimulates L-AA during the LSC process, it is oxidized to l-dehydroascorbic acid and releases two electrons (Figure 2b) [16]. These two electrons generated from the oxidation of L-AA contribute to reducing the GO surface [16]. Therefore, if the LSC process of GO is performed after 15 min of heat treatment in the presence of L-AA, the reduction effect of GO by the electrons released during the oxidation of L-AA can be improved. Therefore, if the LSC process of GO is performed in the presence of L-AA, the graphene micro-pattern surface’s deoxygenation can be promoted. It was confirmed that the micro-patterns of 16 electrodes fabricated through our work were successfully fabricated, and it was observed that the patterned parts became noticeably darker than the non-patterned parts (Figure 2a). This indicates that the LSC process, accompanied by heat treatment and L-AA, effectively reduces GO to RGO in making micro-patterns [11,12,13,14,15]. This study adopted an interdigitated microelectrode type in which two patterns are interlocked on one substrate. In such an interdigitated micro-pattern type, since the movement/exchange of electrons and electrolyte ions becomes smoother in the same substrate, it is more efficient than the conventional sandwich method in improving the performance of the MSC device [11,12,13,14,15]. As-prepared micro-patterns were applied as interdigitated electrodes in MSCs, and the following paragraphs will discuss the effects of the degree of reduction of the micro-patterns on their spectroscopic, electrochemical, and mechanical properties.

**Figure 3:**
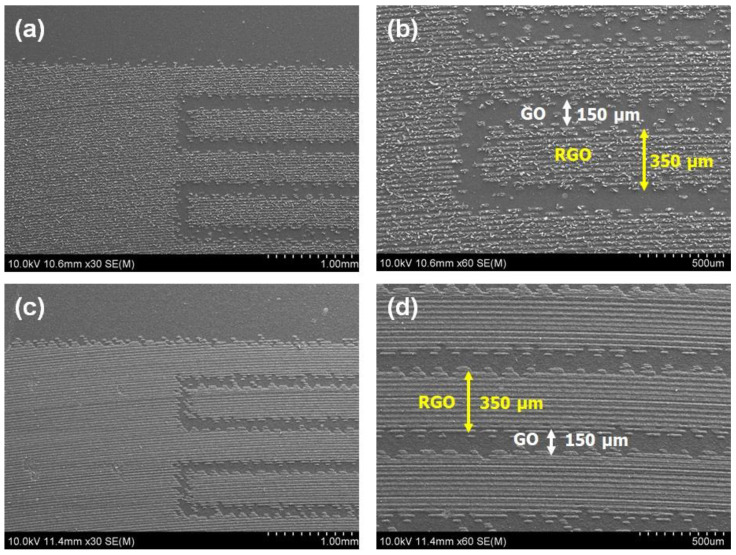
FE-SEM images of graphene micro-patterns manufactured by the LSC process (1 time) obtained at different magnifications: (**a**) ×30 and (**b**) ×60. FE-SEM images of graphene micro-patterns manufactured by the LSC process (5 times) obtained at different magnifications: (**c**) ×30 and (**d**) ×60. Every GO was pretreated with 0.6 M L-AA and heat treatment for 15 min before LSC.

Figure 3 shows the FE-SEM images of the graphene micro-pattern obtained after the LSC process was performed one and five times. Even through one time of LSC, it was successful in fabricating an interdigitated micro-pattern with an electrode width of 350 μm and an inter-electrode spacing of 150 μm (Figure 3a,b). However, the connectivity between the RGO lines inside an electrode remained low (Figure 3b). As a result of increasing the time of LSC to five times, the width of the electrode and the distance between the electrodes were kept constant, while the connectivity between the RGO lines constituting one electrode was remarkably improved (Figure 3c,d). The FE-SEM images presented in Figure S1 offer a detailed overview of how the morphology of the graphene micro-pattern surface is affected by different LSC treatment times. As the number of LSG treatments increased from nought to five, RGO lines began to form, and the gap between them gradually decreased (Figure S1a–f, see Supplementary Materials). After the five times of LSC treatments, the connectivity between RGO lines reached its maximum (Figure S1e, see Supplementary Materials). Beyond the five times of LSC treatments, no further changes were observed in the connectivity between RGO lines (Figure S1f, see Supplementary Materials). The improved connectivity between the RGO lines inside the electrode means a decrease in contact resistance due to the improvement of the conductive channels inside the micro-pattern. Therefore, our work determined that the optimal number of printing times to maximize electrical properties while maintaining a constant resolution of micro-patterns was five times.

**Figure 4:**
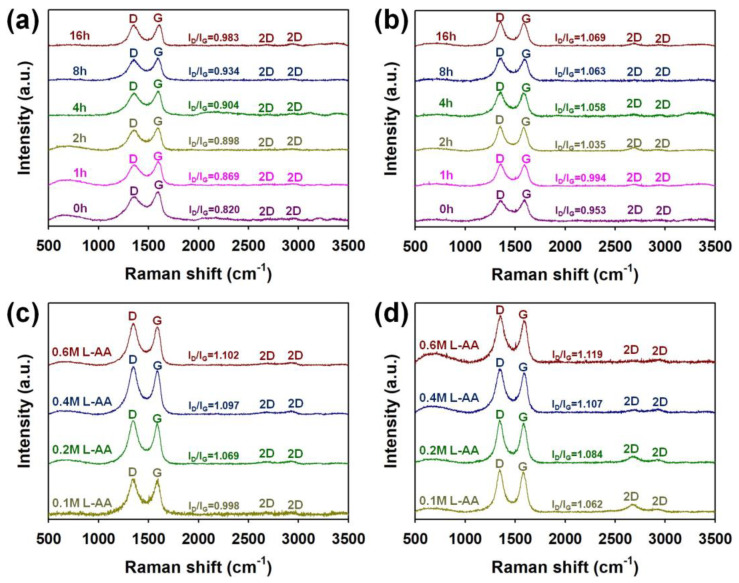
Raman spectra of graphene micro-patterns fabricated (**a**) before and (**b**) after performing the LSC (5 times) of pretreated graphene under different heat-treatment times. Raman spectra of graphene micro-patterns fabricated (**c**) before and (**d**) after performing the LSC (5 times) of graphene pretreated with different molarity of L-AA.

**Figure 5:**
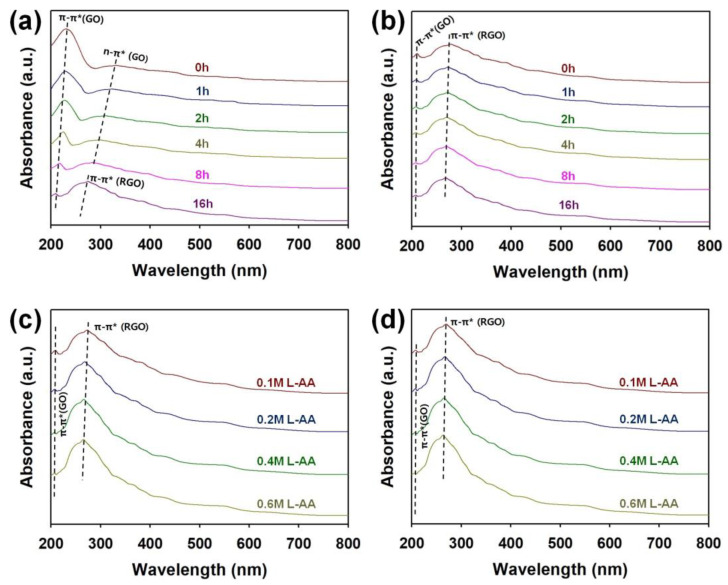
UV–visible spectra of graphene micro-patterns fabricated (**a**) before and (**b**) after performing the LSC (5 times) of pretreated graphene under different heat-treatment times. UV–visible spectra of graphene micro-patterns fabricated through (**c**) before and (**d**) after performing the LSC (5 times) of graphene pretreated with different molarity of L-AA.

**Figure 6:**
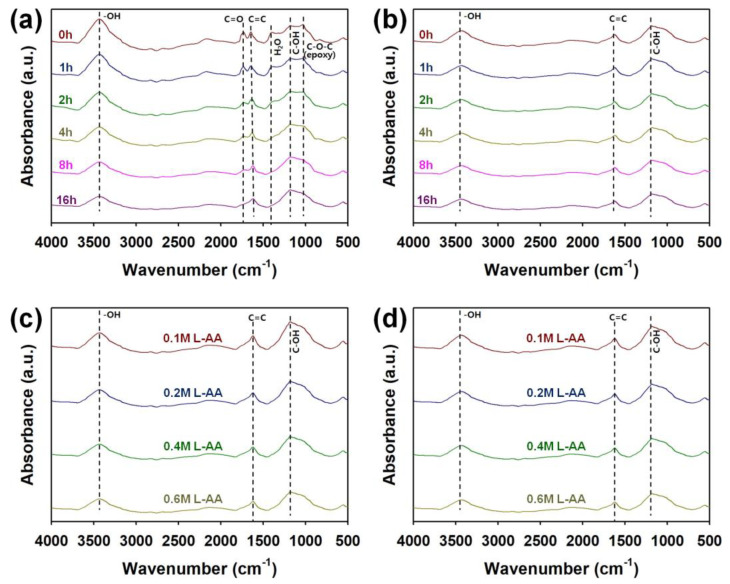
FT-IR spectra of graphene micro-patterns fabricated (**a**) before and (**b**) after performing the LSC (5 times) of pretreated graphene under different heat-treatment times. FT-IR spectra of graphene micro-patterns fabricated through (**c**) before and (**d**) after performing the LSC (5 times) of graphene pretreated with different molarity of L-AA.

**Figure 7:**
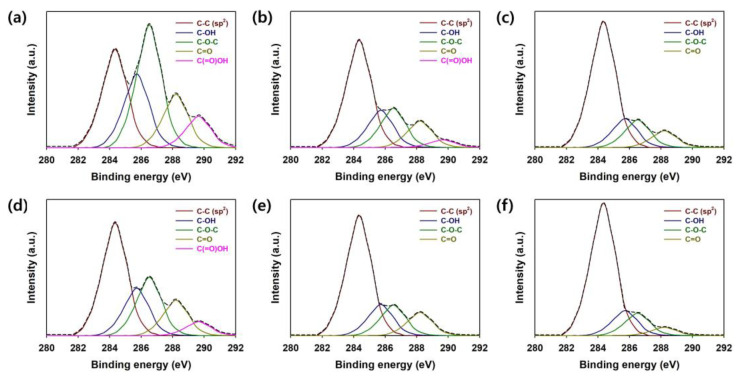
C1s XPS spectra of graphene preheated for 0 h (**a**) before and (**d**) after LSC performing LSC (5 times). C1s XPS spectra of graphene preheated for 16 h (**b**) before and (**e**) after LSC performing LSC (5 times). C1s XPS spectra of graphene pretreated with 0.6 M L-AA (**c**) before and (**f**) after LSC performing LSC (5 times).

In order to identify the effects of differences in heat-treatment time and L-AA concentration on the degree of reduction of graphene through the LSC process, Raman, UV-vis, FT-IR, and XPS spectra were measured (Figure 4, Figure 5, Figure 6 and Figure 7). Figure 4 shows the Raman spectra of graphene micro-patterns obtained under different heat-treatment times, L-AA concentrations, and times of LSC. It was confirmed that as the heat-treatment time of GO without ascorbic acid was increased from 0 to 16 h, the intensity ratio of the D peak to the G band (*I*_D_/*I*_G_) increased (Figure 4a). This is because the degree of reduction of *sp*^3^ orbitals to *sp*^2^ orbitals in graphene increases as the heat-treatment time increases, and structural defects and holes in graphene increase [17,18,19,20]. In addition, it was confirmed that the D/G ratio further increased after conducting LSC (five times) of the heat-treated samples (Figure 4b). Figure 4c shows the Raman spectra of the samples heat-treated for 15 min in the presence of a different molarity of L-AA, and it was confirmed that the D/G ratio slightly increased as the molarity of L-AA increased. Interestingly, the degree of reduction of the sample heat-treated for 15 min in the presence of 0.6 M L-AA was higher than that of the sample heat-treated for 16 h. This suggests that the presence of L-AA significantly shortens the heat-treatment time. The further increase in the D/G ratio after performing LSC (five times) indicates that L-AA contributed to the additional reduction of graphene by the stimulation of the laser inside the DVD burner (Figure 4d). The above results confirmed that heat treatment for 15 min in the presence of 0.6 M L-AA was an optimized pretreatment condition to promote the reduction of GO through the LSC process.

Figure 5 shows the UV–visible spectra of graphene micro-patterns obtained under different heat-treatment times, L-AA concentrations, and times of LSC. As the heat-treatment time increased, the absorption peaks corresponding to the π–π* electron transition of GO and the n–π* electron transition of GO moved to shorter wavelengths and decreased in intensity (Figure 5a) [17,21]. Conversely, the intensity of the absorption peak corresponding to the π–π* electron transition of reduced graphene oxide (RGO) gradually increased with longer heat treatment, indicating that a higher degree of reduction leads to the removal of more oxygen functional groups, decreasing the oxygen atoms that can cause the n–π* electron transition [17,21]. After five times of LSC process, the intensity of the peaks increased only slightly (Figure 5b). As a result of reducing the heat-treatment time to 15 min and increasing the content of L-AA, it was confirmed that the blue shift of the absorption peak corresponding to the π–π* electron transition of RGO and the increase in absorbance intensity became more prominent (Figure 5c). Even after five times of LSC processes, the increase in the peaks corresponding to RGO was minimal, suggesting that the reduction effect by the heat-treatment process accompanied by L-AA before LSC was significant (Figure 5d).

FT-IR spectra of graphene micro-patterns obtained under different heat-treatment times, L-AA concentrations, and time of LSC are shown in Figure 6. The characteristic peaks of graphene were found at 1046, 1188, 1410, 1646, 1730, and 3449–3600 cm^‒1^, and these peaks are attributable to C–O–C stretching of epoxy, C–OH stretching of phenol, O–H stretching of H_2_O, C=C stretching, C=O stretching, and O–H stretching, respectively [17,20]. As the heat-treatment time increased, the intensity of the stretching vibration peaks of the bonds containing oxygen atoms decreased significantly (Figure 6a). In addition, the LSC treatment resulted in disappearance of peaks corresponding to carbonyl groups, water, and epoxy bonds (Figure 6b). When heat treatment was carried out in the presence of L-AA for 15 min, peaks corresponding to C=O, H_2_O, and C–O–C bonds became indistinctive, and as the content of L-AA increased, the intensity of peaks corresponding to C–OH, C=C, and O–H bonds decreased significantly (Figure 6c). After performing the LSC process (five times) on the samples heat treated for 15 min in the presence of L-AA, the change in intensity of peaks corresponding to C-OH, C=C, and O-H bonds was not significant (Figure 6d). This indicates that the reduction of GO was mostly completed in the step of heat treatment for 15 min in the presence of L-AA before LSC, as evidenced in Raman and UV–visible spectra. Through Raman, UV, and FT-IR analyses, it was found that LSC in the presence of L-AA is more effective in increasing the degree of reduction of graphene than the heat treatment for a long time.

The C1s XPS spectra of the graphene samples prepared under different conditions were studied to analyze the bonding structure of the prepared graphene samples (Figure 7 and Table 1). The oxygenated carbon groups in pristine GO (0 h heating) accounted for 74.2% of the total, and the *sp*^2^-hybridized C=C bond accounted for only 25.8%. This indicates that most of the carbons that make up pristine GO are *sp*^3^-hybridized (Figure 7a and Table 1) [10,11,12,13,14,15,17,21]. It was confirmed that the proportion of *sp*^2^-hybridized C=C bonds contained in the sample preheated with GO for 16 h and the sample with GO preheated under 0.6 M L-AA for 15 min increased to 49.3 and 63.3%, respectively (Figure 7a,b and Table 1). Furthermore, it was found that the carboxylic acid groups completely disappeared after heat treatment of graphene in the presence of 0.6 M L-AA for 15 min. These results demonstrate that the reduction effect using L-AA is much more efficient than GO reduction through heat treatment for 16 h [10,11,12,13,14,15,17,21]. After five times of LSG treatments, the proportion of *sp*^2^-hybridized C=C bonds contained in graphene materials increased as follows: 0 h heating (42.1%) < 16 h heating (58.6%) < 0.6 M L-AA (70.4%) (Figure 7d–f and Table 1). Considering the above facts, it was possible to prove through XPS C1s spectra that the presence of 0.6 M L-AA was very effective in improving the reduction effect through the LSG process, and it perfectly matched the tendency of the Raman spectra shown in Figure 4 [10,11,12,13,14,15,17,21].

**Figure 8:**
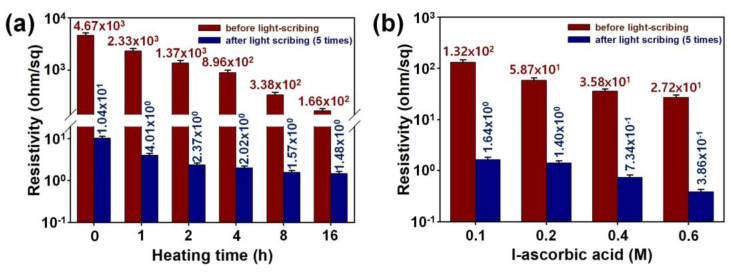
(**a**) Surface resistivity of graphene micro-patterns fabricated before and after performing LSC (5 times) of pretreated graphene under different heat treatment times. (**b**) Surface resistivities of graphene micro-patterns fabricated before and after performing LSC (5 times) of pretreated graphene under different heat treatment times.

In order to identify the effects of heat-treatment time, L-AA concentration, and LSC on the electrical properties of graphene micro-patterns, the surface resistivities of the samples are summarized in Figure 7. As the heat-treatment time increased, the surface resistance of graphene significantly decreased, and the surface resistance of the sample heat-treated for 16 h (1.66 × 10^2^ Ω/sq) was reduced by 28.1 times compared to pristine GO (4.67 × 10^3^ Ω/sq) (Figure 8a). In addition, when LSC (five times) of the sample heat-treated for 16 h was performed, it was found that the surface resistance decreased by 112.2 times (1.4867 × 10^0^ Ω/sq) compared to before the LSC process (1.6667 × 10^2^ Ω/sq). Furthermore, when LSC was performed on the sample heat-treated for 16 h, the surface resistance decreased by 112.2 times (1.4867 × 10^0^ Ω/sq). The LSC process was found to be highly effective in enhancing the degree of GO reduction, leading to significantly improved electrical properties of graphene micro-patterns [10,11,12]. The surface resistivities of the graphene samples treated with heat for 15 min in the presence of L-AA were 10 to 100 times lower than those subjected to heat treatment for a long time without L-AA (Figure 8b). Notably, the resistance of graphene pretreated with 0.6 M L-AA (2.7267 × 10^1^ Ω/sq) was 171.7 times lower than that of pure GO, and this decreased further by 6.10 times (3.8667 × 10^−1^ Ω/sq) after the LSC process was performed 5 times. The involvement of L-AA in the reduction of GO during the LSC process was found to restore the *sp*^2^-hybridized carbon structures and expand the conjugated system structure, thereby promoting the delocalization of electrons [10,11,12]. Considering these results, pretreating GO through heat treatment for 15 min in the presence of 0.6 M L-AA is more effective in enhancing the LSC efficiency than pretreating GO through heat treatment for 16 h, facilitating the production of graphene micro-patterns with improved electrical properties.

**Figure 9:**
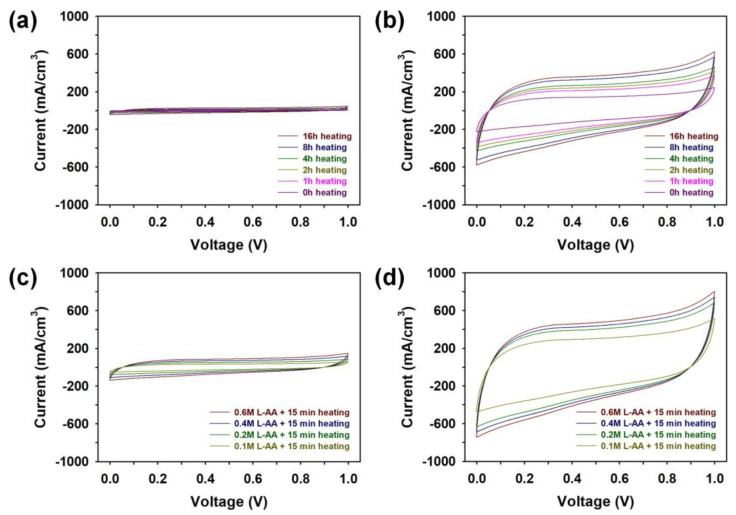
Cyclic voltammograms (CVs) of MSCs fabricated (**a**) before and (**b**) after performing LSC process (5 times) of pretreated graphene under different heat-treatment times. CVs of MSCs fabricated through (**c**) before and (**d**) after performing LSC process (5 times) of graphene pretreated with different molarity of L-AA. These CV curves were measured at a scan rate of 10 mV/s.

In order to identify the effects of heat-treatment time, L-AA concentration, and LSC process on the electrochemical activity of graphene micro-patterns, the CVs of MSCs employing graphene micro-patterns prepared under different manufacturing conditions were measured at a scan rate of 10 mV/s (Figure 9). All CV curves exhibited typical rectangular responses, suggesting that the graphene micro-patterns store electrical charge well, according to the EDLC mechanism [10,11,12,13,14,15]. As the duration of heat treatment increased, the CV curves of graphene also increased in size, and after the LSC process, these curves became significantly larger (Figure 9a,b). These findings confirm that both heat treatment and the LSC process enhance the reduction of graphene, ultimately leading to improved electrochemical performance. Moreover, compared to samples prepared by heat treatment without L-AA, those treated with L-AA for 15 min exhibited much larger CV curves (Figure 9c). Increasing the L-AA content led to higher CV peaks, signifying that L-AA plays a substantial role in reducing graphene and boosting electrochemical activity [16]. Additionally, micro-patterns produced through the LSC process of samples pretreated with L-AA exhibited even more larger CV curves (Figure 9d). Consequently, the pretreatment of GO with heat treatment in the presence of 0.6 M L-AA for 15 min facilitated better charge transport in the interdigitated graphene micro-patterns, resulting in enhanced electrochemical performance of the samples. After analyzing Raman, FT-IR, UV–vis, and XPS spectra, it became evident that the most effective concentration of L-AA for maximizing the reduction efficiency of graphene oxide (GO) was 0.6 M. The reduction process using 0.6 M L-AA demonstrated significantly superior efficiency compared to long-term heat treatment without L-AA (Tables S1 and S2, see Supplementary Materials). Conducting LSC in the presence of 0.6 M L-AA facilitated the realization of the best electrical and electrochemical properties (Table S2, see Supplementary Materials). Therefore, our work concluded that the optimal concentration of L-AA to maximize the reduction effect by LSC was determined to be 0.6 M.

**Figure 10:**
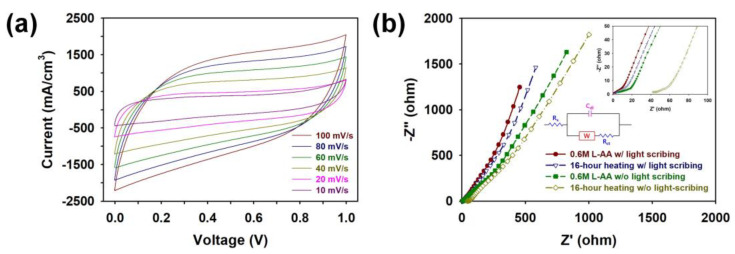
(**a**) CV curves of an MSC containing a graphene micro-pattern prepared by the LSC process (5 times) in the presence of 0.6 M L-AA measured at different scan rates. (**b**) Nyquist impedance plots of MSCs containing graphene micro-patterns prepared under different manufacturing conditions in the frequency range from 1 MHz to 10 mHz.

The LSC micro-patterns prepared by heat treatment of 15 min in the presence of 0.6 M L-AA showed rectangular CV curves at various scan rates from 10 to 100 mV/s, indicating that the MSCs fabricated through this study can perform well as an energy storage device at various scan rates (Figure 10a). In order to understand the factors hindering current flow within the MSCs, we measured the Nyquist plot of each device using Electrochemical Impedance Spectroscopy (EIS) (Figure 10b). To explain the stability of the micropatterned electrodes, the EIS plots fitted using a suitable circuit model are shown in Figure 10b (inset). The initial intercept at the real axis in the Nyquist plots represent the series resistance (*R*_s_), resulting from the combination of electrolyte resistance and intrinsic resistance of graphene micro-patterns [10,11,12,13,14,15]. The *R*_s_ of the MSCs decreased in the following order: 16 h heating without LSC (42.1 Ω) > 0.6 M L-AA without LSC (5.18 Ω) > 16 h heating with LSC (2.48 Ω) > 0.6 M L-AA with LSC (0.88 Ω). The second part of the Nyquist plots of the samples relates to the combination of charge transfer resistance (*R*_ct_) and double-layer capacitance (*C*_dl_) at the electrode/electrolyte interface [10,11,12,13,14,15]. The *R*_ct_ of the MSCs decreased in the following order: 16 h heating without LSC (57.3 Ω) > 0.6 M L-AA without LSC (18.2 Ω) > 16 h heating with LSC (15.1 Ω) > 0.6 M L-AA with LSC (10.7 Ω). These results suggest that the LSC process in the presence of L-AA effectively reduced impedance in the MSC device. Vertical straight lines were observed in the low-frequency region in every Nyquist plot, demonstrating that the interdigitated graphene micro-patterns facilitate efficient ion diffusion and appropriate capacitive behavior [10,11,12,13,14,15].

**Figure 11:**
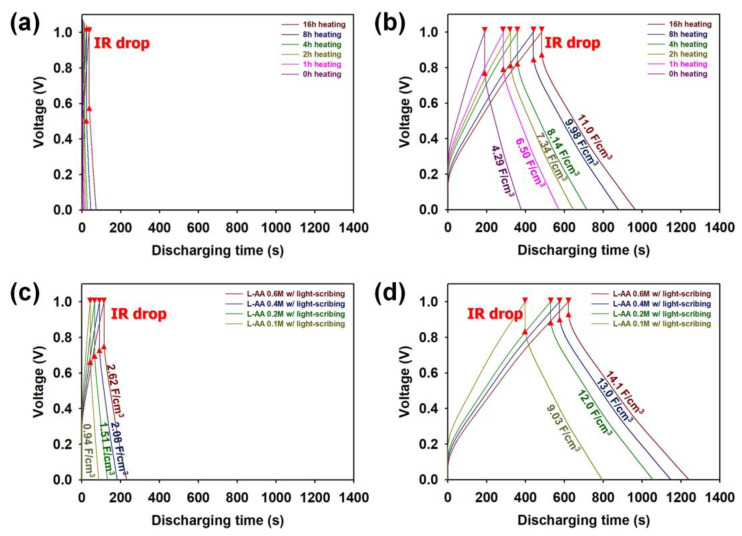
Galvanostatic charge–discharge (GCD) curves of MSCs fabricated (**a**) before and (**b**) after performing the LSC process (5 times) of pretreated graphene under different heat-treatment times. GCD curves of MSCs fabricated (**c**) before and (**d**) after performing the LSC process (5 times) of graphene pretreated with different molarity of L-AA. These GCD curves were measured at a current density of 0.10 A/cm^3^.

In order to evaluate the capacitive performances of MSCs employing graphene micro-patterns prepared under different manufacturing conditions, GCD curves were measured at a current density of 0.10 A/cm^3^ with a voltage from 0 to 1.0 V (Figure 11). Every GCD curve exhibited a symmetrical shape, indicating that the interdigitated MSCs enable high Coulombic efficiency. The internal resistance (IR) of the MSCs was estimated from the voltage drop at the onset of the GCD curves [10,11,12,13,14,15,22]. The IRs measured from the GCD curves were consistent with the Raman, FT-IR, UV–vis, XPS, surface resistance, CV, and EIS results. These results reconfirm that the LSC process in the presence of L-AA is highly effective in improving the electrical conductivity of graphene micro-patterns. The discharging time of the GCD curve of the MSCs increased as the heating time increased, and the increase in the discharging time of the GCD curve became more significant (Figure 11a,b). These results reconfirm that the heat treatment and LSC processes enhance the capacitive behaviors of graphene micro-patterns. As the L-AA content increased, the discharging time increased, indicating that the presence of L-AA significantly contributed to the increase in the storage capacity of the graphene micro-pattern (Figure 11c). In particular, the micro-pattern obtained through the LSC process of the sample pretreated with 0.6 M L-AA exhibited the maximum volumetric capacitance of 14.1 F/cm^3^, indicating its improved capacitive performance compared to the LSC-based MSCs reported in the recent work (Figure 11d) [10,11,12,13,14,15]. Our findings demonstrate that the modified LSC process can significantly improve the reduction efficiency of GO and the capacitive performance of the MSC in the presence of the optimized molarity of L-AA (Table S3, see Supplementary Materials).

**Figure 12:**
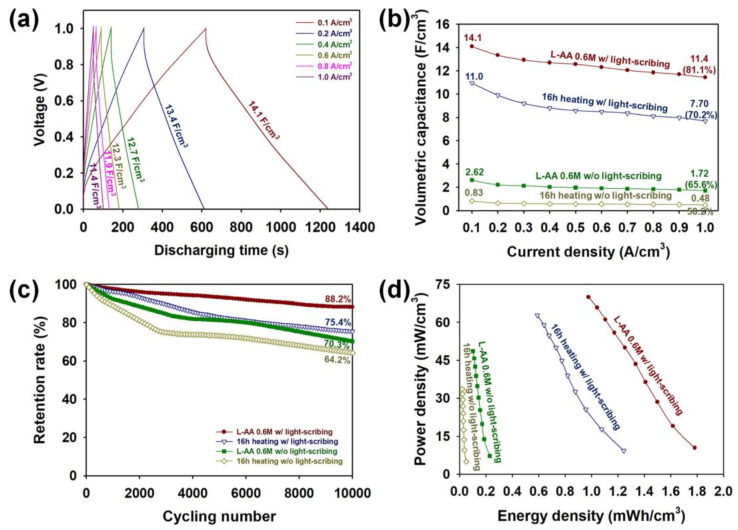
(**a**) GCD curves of an MSC device containing a graphene micro-pattern prepared by the LSC process (5 times) in the presence of 0.6 M L-AA measured at different current densities. (**b**) Rat- capability curves of MSCs containing graphene micro-patterns prepared under different manufacturing conditions at different current densities. (**c**) Cycling stability of MSCs containing graphene micro-patterns prepared under different manufacturing conditions after 10,000 charge/discharge cycles at a current density of 0.40 A/cm^3^. (**d**) Ragone plots of MSCs containing graphene micro-patterns prepared under different manufacturing conditions.

Figure 12a shows GCD curves of the MSCs prepared by heat treatment of 15 min in the presence of 0.6 M at various current densities from 0.10 to 1.00 A/cm^3^, and the retention rates at 0.20, 0.40, 0.60, 0.80, and 1.00 A/cm^3^ compared to the initial retention rate obtained at 0.10 A/cm^3^ were 95.0, 90.0, 87.2, 84.4, and 81.1, respectively. In addition, it was evident that the L-AA 0.6 M sample demonstrated higher rate capability than the heating 16 h sample, and the rate capability of the sample became significantly higher after the LSC process (Figure 12b). This implies that the LSC process**,** in the presence of optimized L-AA content**,** effectively retards the inevitable capacitance losses at higher currents [10,11,12,13,14,15]. We investigated the long-term cycling stability of the graphene MSCs by performing 10,000 charge/discharge cycles at a current density of 0.40 A/cm^3^ (Figure 12c). The retention rate of the L-AA 0.6 M sample (70.3%) was higher than the heating 16 h sample (64.2%), indicating that the pretreatment of GO through heat treatment for 15 min in the presence of 0.6 M L-AA was more effective in reducing the oxygenated functionalities of GO than the pretreatment of GO through heat treatment for 16 h. After the LSC process (five times), the retention rate of the L-AA 0.6 M and heating 16 h samples was 88.2 and 75.4%, respectively. This demonstrates that the presence of 0.6 M L-AA in the LSC process is more efficient in suppressing the undesirable shrinkage and delamination of the graphene sheets during the repetitive charge/discharge processes [10,11,12,13,14,15]. Furthermore, the GCD results also suggest that the LSC process allows the electrolytes to penetrate deeply into the underlying layers of the graphene micro-patterns [12]. To further examine the practical applicability of the MSCs, Ragone plots of the samples employing graphene micro-patterns prepared under different manufacturing conditions are summarized in Figure 12d. The maximum volumetric energy densities (given in mWh/cm^3^) of the MSCs increased in the following order: 16 h heating without LSC (0.05) < 0.6 M L-AA without LSC (0.23) < 16 h heating with LSC (1.24) < 0.6 M L-AA with LSC (1.78). The maximum power densities (given in mW/cm^3^) of the MSCs increased in the following order: 16 h heating without LSC (33.7) < 0.6 M L-AA without LSC (48.6) < 16 h heating with LSC (62.7) < 0.6 M L-AA with LSC (69.9). Table 2 summarizes the electrochemical performance of recent studies on light-scribed graphene (LSG) for MSCs. The electrochemical performance of the MSC obtained through this study showed superior capacitance and energy density per volume compared to previous research results. The modified LSC process proposed by our work is highly effective in improving the energy/power output of MSCs and enables facile control of the desired energy/power output [10,11,12].

## 4. Conclusions

A facile and effective LSC process to obtain graphene micro-patterns with high conductivity and excellent electrochemical activity has been developed successfully and applied to manufacture high-performance MSCs. The LSC process of GO in the presence of photosensitive L-AA not only improved the degree of reduction of graphene micro-patterns but also significantly shortened the preheating time to obtain highly conductive micro-patterns. By performing the LSC process accompanied by a 15 min preheat treatment of GO in the presence of 0.6 M L-AA, we were able to achieve the highest degree of reduction of graphene micro-patterns, as demonstrated in the Raman, UV–visible, FT-IR, and XPS spectra. Surface resistance, CV, EIS, and GCD results also followed the same trend as the spectroscopic analysis results, proving that this LSC process ultimately contributed to improving the electrical and electrochemical performances of micro-patterns. The fabricated LSC-MSC demonstrated an outstanding volumetric capacitance of 14.1 F/cm^3^, a high energy density of 1.78 mWh/cm^3^, and a high power density of 69.9 mW/cm^3^. The LSC-MSC also exhibited excellent cycling stability (88.2% after 10,000 cycles) and high rate capability (81.1% at a current density of 1.00 A/cm^3^). This research provides a facile and effective LSC process to fabricate high-performance graphene micro-patterns with greatly improved electrical and electrochemical properties. Our work not only inspires the designs of interdigitated MSC, but also accelerates the development of various miniaturized electronic devices.

## Figures and Tables

**Table 1:** The intensity ratio of peaks in the C1s XPS spectra and the intensity ratio of D band to G band (D/G ratio) of graphene materials.

Sample	^1^ C=C (*sp*^2^) %	^1^ C-OH %	^1^ C-O-C %	^1^ C=O %	^1^ C(=O)OH %	^2^ *I*_D_/*I*_G_
0-h heating w/o LSC	25.8	19.5	32.3	14.0	8.26	0.820
0-h heating w/LSC	42.1	17.7	21.8	13.2	5.17	0.953
16-h heating w/o LSC	49.3	17.1	18.0	12.1	3.50	0.983
16-h heating w/LSC	58.6	15.2	14.9	11.3	-	1.069
0.6 M L-AA w/o LSC	63.3	14.5	13.9	8.35	-	1.102
0.6 M L-AA w/LSC	70.4	13.3	11.9	4.38	-	1.119

^1^ The proportion of the *sp*^2^-hybridized C=C bond was determined through C1s XPS analysis. ^2^ The intensity ratio of the D peak to the G band (*I*_D_/*I*_G_) was calculated from Raman spectroscopy.

**Table 2:** Comparison of electrochemical performance of recent studies on LSG for MSCs.

Electrode Materials	C_A_ (mF/cm^2^)	C_F_ (F/cm^3^)	*E*_max_ (mWh/cm^3^)	*P*_max_ (mW/cm^3^)	Capacitance Retention (%)	Cycles	Ref.
LSG	4.82	-	1.36	20,000	96.5	10,000	[10]
LSG	2.32	2.35	-	200,000	97	2000	[11]
LSG-PANI		4.60	0.407	196	90	10,000	[12]
ZnO/LSG	-	3.90	0.43	40	70	10,000	[13]
Co/LSG	-	2.27	1.06	97	70	10,000	[14]
Co-Ni/LSG	-	5.50	0.63	54	90	3000	[15]
This work	-	14.1	1.78	69.9	88.2	10,000	-

## Data Availability

The original contributions presented in the study are included in the article, further inquiries can be directed to the corresponding authors.

## Supplementary Materials

The following supporting information can be downloaded at https://www.mdpi.com/article/10.3390/mi15070858/s1, Figure S1. FE-SEM images of graphene micro-patterns manufactured by different times of the LSC process (magnification: ×1.00 k), Table S1. The effects of heating time on the reduction efficiency, electrical, and electrochemical properties of graphene materials, Table S2. The effects of L-AA concentration on the reduction efficiency, electrical, and electrochemical properties of graphene materials, Table S3. Summary of spectroscopic, electrical and electrochemical properties of graphene micro-patterns prepared under different manufacturing conditions.
