# Supplementary material for: Development of Light-Scribing Process Using L-Ascorbic Acid for Graphene Micro-Supercapacitor
Source: Micromachines (Basel). 2024 Jun 30;15(7):858. doi: 10.3390/mi15070858 (PMC11279208; doi:10.3390/mi15070858)
Supplement: Supplementary file 1 [file micromachines-15-00858-s001.zip › micromachines-2915835-supplementary.pdf]

micromachines

MDPI

Article

Development of Light-Scribing Process Using L-Ascorbic Acid for Graphene Micro-Supercapacitor

Seorin Park, Da Young Lee and Sunghun Cho *

School of Chemical Engineering, Yeungnam University, Gyeongsan 38541, Republic of Korea
* Correspondence: shcho83@ynu.ac.kr; Tel.: +82-53-810-2535

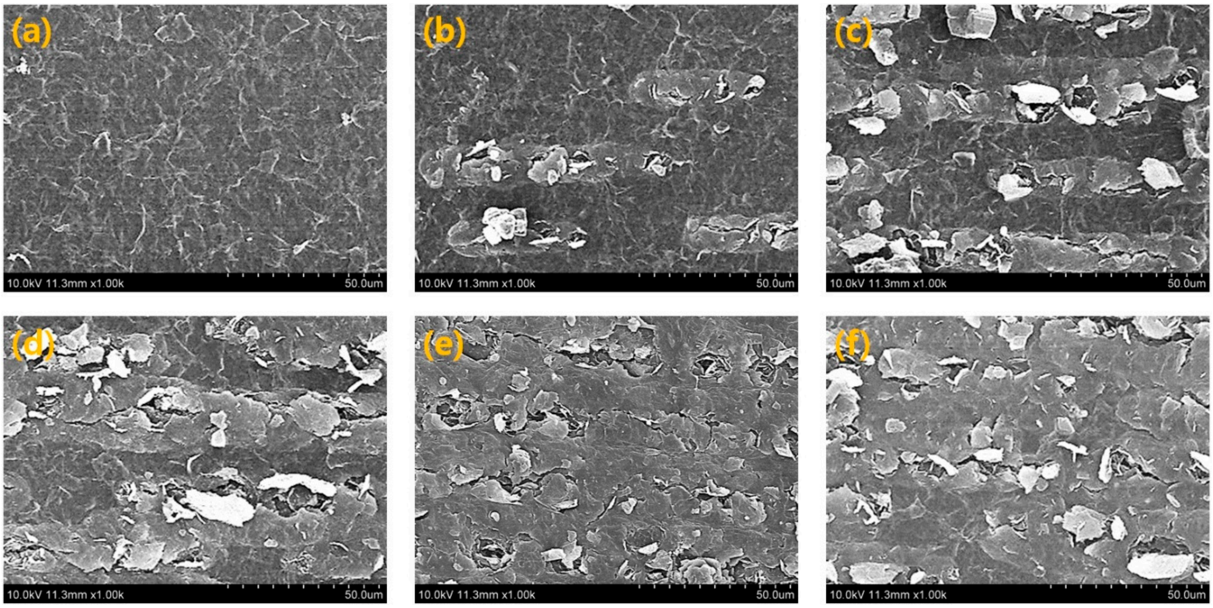

Figure S1. FE-SEM images of graphene micro-patterns manufactured by different times of the LSC process (magnification: x1.00k): (a) 1, (b) 2, (c) 3, (d) 4, (e) 5, and (f) 6.

Table S1. The effects of heating time on the reduction efficiency, electrical, and electrochemical properties of graphene materials.

Heating time (hour)	[1] I_D/I_G		[2] R (Ω/sq)		[3] IR (Ω/cm³)		[4] Δt (sec)		[5] C_v (F/cm³)	
	w/o LSC	w/ LSC	w/o LSC	w/ LSC	w/o LSC	w/ LSC	w/o LSC	w/ LSC	w/o LSC	w/ LSC
0	0.820	0.953	4670	10.4	33.4	8.35	3.43	188.7	0.0779	4.29
1	0.869	0.994	2330	4.01	31.0	7.70	5.77	285.9	0.131	6.50
2	0.898	1.035	1370	2.37	26.9	7.04	9.19	323.0	0.209	7.34
4	0.904	1.058	896	2.02	24.0	6.35	13.6	358.1	0.308	8.14
8	0.934	1.063	338	1.57	18.9	4.96	22.6	439.2	0.513	9.98
16	0.983	1.069	166	1.48	14.9	4.24	36.4	482.7	0.826	11.0

[1] The intensity ratio of the D peak to the G band was determined through Raman spectroscopy.
[2] The surface resistivity was measured using a 4-point probe conductivity meter.
[3] The internal resistance was calculated from GCD curves.
[4] The discharging time was calculated from GCD curves.
[5] The volumetric capacitance was calculated from GCD curves.

Table S2. The effects of L-AA concentration on the reduction efficiency, electrical, and electrochemical properties of graphene materials.

L-AA concentration (M)	[1]I_D/I_G		[2]R (Ω/sq)		[3]IR (Ω/cm³)		[4]Δt (sec)		[5]C_v (F/cm³)	
	w/o LSG	w/ LSG	w/o LSG	w/ LSG	w/o LSG	w/ LSG	w/o LSG	w/ LSG	w/o LSG	w/ LSG
0.1	0.998	1.062	132	1.64	12.2	0.129	43.8	397.5	0.94	9.03
0.2	1.069	1.084	58.7	1.40	11.3	0.0798	66.3	528.1	1.51	12.0
0.4	1.097	1.107	35.8	0.734	10.0	0.0630	91.4	574.0	2.08	13.0
0.6	1.102	1.119	27.2	0.386	8.99	0.0458	115.1	620.6	2.62	14.1

[1] The intensity ratio of the D peak to the G band was determined through Raman spectroscopy.
[2] The surface resistivity was measured using a 4-point probe conductivity meter.
[3] The internal resistance was calculated from GCD curves.
[4] The discharging time was calculated from GCD curves.
[5] The volumetric capacitance was calculated from GCD curves.

Table S3. Summary of spectroscopic, electrical and electrochemical properties of graphene micropatterns prepared under different manufacturing conditions.

Sample	[1]C=C (sp^2) %	[2]I_D/I_G	[3]R_s (Ω)	[4]R_{ct} (Ω)	[5]C_v (F/cm³)	[6]% at 1.00 A/cm³	[7]% after 10,000 cycles	[8]E_{max} (mWh/cm³)	[9]P_{max} (mW/cm³)
16-hour heating w/o LSC	49.3	0.983	42.1	57.3	0.826	58.2	64.2	0.05	33.7
16-hour heating w/ LSC	58.6	1.069	2.48	15.1	11.0	70.2	75.4	1.24	62.7
0.6 M L-AA w/o LSC	63.3	1.102	5.18	18.2	2.62	65.6	70.3	0.23	48.6
0.6 M L-AA w/ LSC	70.4	1.119	0.88	10.7	14.1	81.1	88.2	1.78	69.9

[1] The proportion of the sp^2-hybridized C=C bond was determined through C1s XPS analysis.
[2] The intensity ratio of the D peak to the G band was determined through Raman spectroscopy.
[3] The series resistance was determined through EIS analyses.
[4] The charge transfer resistance was determined through EIS analyses.
[5] The volumetric capacitance was calculated from GCD curves.
[6] The retention rate at 1.00 A/cm³ was determined through GCD analyses.
[7] The cycling stability after 10,000 charge/discharge cycles was determined through GCD analyses.
[8] The volumetric energy density was calculated as $E = C_v \Delta V^2/2$.
[9] The volumetric power density was calculated as $P = E/t$.
